# A questionnaire to measure melanoma risk, knowledge and protective behaviour: Assessing content validity in a convenience sample of Scots and Australians

**DOI:** 10.1186/1471-2288-11-123

**Published:** 2011-08-25

**Authors:** Helen S Gillespie, Tony Watson, Jon D Emery, Amanda J Lee, Peter Murchie

**Affiliations:** 1 Centre of Academic Primary Care, University of Aberdeen, Polwarth Building, Foresterhill, Aberdeen, AB25 2ZD, Scotland; 2 School of Primary, Aboriginal and Rural Health Care, The University of Western Australia (M501), 35 Stirling Highway, Crawley, 6009, Western Australia

## Abstract

**Background:**

The aim of this study was to assess the content validity of a questionnaire to measure melanoma risk, knowledge and protective behaviour in a convenience sample of Scots and Australians. Australia has the highest melanoma incidence worldwide but has developed a culture of skin cancer avoidance with a long history of skin cancer primary prevention campaigns of proven effectiveness. Scotland has lower incidence, but has shown a greater rate of increase between 1985 and 2007. There is an urgent need in Scotland, therefore, to identify those groups at greatest risk and provide them with effective preventative advice.

**Method:**

A self-administered postal survey was completed by four groups formed from convenience samples in two geographical locations (Northeast Scotland and Western Australia). In univariate analysis scores on *personal risk, level of concern, protective behaviour, and knowledge *were compared by nationality, previous skin cancer diagnosis and personally knowing someone with melanoma. Multivariate linear regression analysis modelled the influence of potential predictor variables upon each of the scores.

**Results:**

540 people completed the questionnaire, 273 Scots (50.6%). 133 (24.6%) Scots and 83 (15.4%) Australians previously had melanoma or non-melanoma skin cancer, whilst 120 (22.2%) Scots and 190 (35.2%) Australians personally knew someone with melanoma. Australians had higher knowledge *(p < 0.001)*, level of concern *(p < 0.001) *and protective behaviour *(p < 0.001) *scores than the Scottish. Australian nationality was the strongest independent predictor of a higher knowledge score (*p < 0.001*), followed by a previous skin cancer diagnosis (*p = 0.003*), personal knowledge of someone with melanoma (*p = 0.011*), female gender (*p = 0.005*) and higher education status (*p < 0.001*) *(R^2 ^= 0.163).*

**Conclusion:**

The questionnaire detected higher levels of knowledge and skin cancer protective behaviours in Australians than in Scottish people. This was expected and supports the content validity of the questionnaire and its value as a future research tool in the Scottish population.

## Background

The incidence of cutaneous melanoma is increasing in most Caucasian populations and is viewed as an increasing global public health problem[1,2]. This is particularly so in Scotland where the incidence of melanoma is increasing rapidly, by 85% in men and 31% in women between 1983 and 2001[3,4]. If current trends are maintained Scottish melanoma incidence rates will double every 10-20 years[5]. There is, therefore, a pressing need in Scotland to identify those most at risk of melanoma and provide them with appropriate preventative advice. Current evidence suggests those previously diagnosed with melanoma, those with a family history of melanoma, and young men of lower socioeconomic and educational status may benefit from targeted advice[6-10]. Other factors which could be important, however, such as place of residence and occupation, have yet to be explored in Scotland.

Australia has the highest worldwide incidence of melanoma (48.7 per 100,000 people in 2007, compared to 21.1 per 100,000 in Scotland)[11]. Consequently, efforts to prevent melanoma based on sun protection began in the 1960s, much earlier than elsewhere in the world[12]. Co-ordinated nationwide campaigns began with Slip! Slap! Slop! in 1980 and were followed by SunSmart, which continues at the current time, particularly emphasising the importance of reducing sun exposure in children[3,13,14]. Following sustained effort over three decades, formal evaluation suggests that such campaigns have contributed to changed cultural attitudes with respect to the sun and protective behaviour, and there is evidence of reduced skin cancer incidence amongst younger people born after 1960, when the campaigns first began[3,13,14].

In contrast melanoma prevention efforts in Scotland have been sporadic and focused on early detection and treatment rather than prevention[3,6]. Since 2003, however, Cancer Research UK in conjunction with the UK government has run the SunSmart campaign, based upon the Australian model[6,7]. This campaign, however, has yet to be formally evaluated and may not represent the best use of resources given the apparent urgency with which preventative behaviour would appear to need to be improved amongst those Scottish people at highest risk. To wait for cultural change to accrue over decades may not be the best way to approach Scotland's dramatically increasing melanoma incidence.

Our aim in conducting this pragmatic study was to assess the content validity of a questionnaire adapted to compare personal risk, knowledge and protective behaviour with respect to melanoma. Content validity has been defined as "whether or not the items sampled for inclusion on the tool adequately represent the domain of content addressed by the instrument"[15]. We did this by comparing responses from a convenience sample of people in Australia and Scotland. If proven valid, we believed that the questionnaire could provide an invaluable research tool in two respects: first, to identify those groups at greatest risk of developing melanoma: and second, to gauge the effect of targeted advice to high risk groups.

## Methods

### Setting

This study was conducted in Northeast Scotland and in Perth, Western Australia.

### Ethical approval

Full ethical approval for the study was granted by the North of Scotland Research Ethics Committee (REC reference number: 08/S0801/13) on February 15 2008, and by the Human Research Ethics Committee of the University of Western Australia in April 2008.

### Participants

The questionnaire was completed by a convenience sample of participants drawn from four population groups which we believed would differ with respect to melanoma risk and knowledge. The first group comprised people from Northeast Scotland that had been treated for melanoma and had participated in a randomised trial of GP-led follow-up for melanoma. The second group comprised people aged between 18 and 80 recruited through three general practices in Northeast Scotland who, according to their primary care-held medical records, had never been diagnosed with any form of skin cancer. The third group comprised people aged between 18 and 80 who attended one of three Perth-based GPs. The fourth group comprised people that had self-referred themselves to a commercial mole screening clinic in Western Australia. Australian people previously affected by melanoma and non-melanoma skin cancers were included in both the third and fourth groups.

### Questionnaire and data collection

The questionnaire had been adapted by us, with permission, from an existing instrument resulting in a self-completion questionnaire comprising 28 questions[16,17]. The first six questions gathered data on demographics and respondents' personal experience of melanoma. The remaining 22 questions collected information on four domains: respondents' personal risk of melanoma (7 questions); their level of concern about melanoma (i.e. how often they checked their own skin and how likely they were to seek medical advice about skin lesions) (4 questions); self-reported behaviour to protect themselves against melanoma (7 questions), and knowledge about melanoma (4 questions). For each of the 22 questions respondents were presented with a range of options, from which they selected one. Each individual response attracted a score. Scores within each domain were then summed to produce four overall scores, personal risk, level of concern, protective behaviour, and melanoma knowledge. The approach used to define the summary scores was intuitive, but grounded in relative effect sizes from epidemiological meta-analyses[18-24]. Because the questionnaire used multiple response items, which varied in the number of responses which people could assign, it would not have been valid to conduct conventional tests of internal consistency. The details of potential responses, the scores associated with each response, the way in which domain scores were calculated and their ranges are all shown in detail in Table 1. The questionnaire was mailed to participants in each of the four groups for self completion.

**Table 1:** Calculation of personal risk, level of concern, protective behaviour and knowledge summary scores from the study questionnaire

Domain		Range of possible scores	Total
			

**PERSONAL RISK**			

**What happens to your skin in sun?**	never tans **(+6)**, tans with difficulty **(+4)**, tans easily **(+2)**, tans always **(0)**	0 to 6	

**Does your skin freckle?**	Yes **(+2) **or no **(0)**	0 to 2	

**How many moles do you have?**	None **(0)**; less than 20 **(+2)**; more than 20 **(+4)**	0 to 4	

**Do you have moles with irregular edge and colour?**	Yes **(+2) **or no **(0)**	0 to 2	

**What is your natural hair colour?**	black **(0)**, brown **(0)**, red **(+2)**, fair **(+1)**	0 to 2	

**What is your ethnic origin**	Caucasian **(+2)**, Asian **(0)**, Afro-carribean **(-2)**, Chinese **(0)**, Other - specify **(0)**	-2 to 2	

**How many times have you had bad sunburn?**	Never **(0)**, once or twice **(+2)**, three or more **(+4)**	0 to 4	**-2 to 22**

			

**LEVEL OF CONCERN**			

**Have you seen GP with a skin lesion in last 6 months?**	Yes **(+2) **or no **(0)**	0 to 2	

**How likely are you to see GP with a skin lesion in next 6 months?**	Very likely **(+4)**, likely **(+2)**, not likely, **(0)**, very unlikely **(0)**	0 to 4	

**Do you check your own skin for moles?**	Yes **(+1) **or no **(0)**	0 to 1	

**If yes, how often?**	More than monthly **(+3)**, monthly **(+2)**, once or twice a year **(+1)**	1 to 3	**1 to 10**

			

**PROTECTIVE BEHAVIOUR**			

**Do you try to get a suntan at home or on holiday?**	Yes **(+1) **or no **(0)**	0 to 1	

**Do you use a sun bed or sun lamp?**	Yes **(+1) **or no **(0)**	0 to 1	

**If you use a sun bed or sum lamp, how often?**	Very infrequently **(0)**, 1-3 times year **(-1)**, monthly **(-2)**, weekly or more **(-3)**	-3 to 0	

**Do you protect your skin at home or on holiday?**	Yes **(+1) **or no **(0)**	0 to 1	

**How many sunny foreign holidays have you had in 5 years?**	None **(+3)**, one or two **(+2)**, three or four **(+1)**, five or more **(0)**	0 to 3	

**What do you do on noticing a new mole?**	Visit my GP **(+2)**, ask partner or friend to look at **(+1)**, ignore it **(0)**	0 to 2	

**How quickly should a new mole be checked?**	< 1 month **(+4)**, < 2 months **(+3)**, <3 months **(+2)**, eventually **(+1)**, never **(0)**	0 to 4	**-3 to12**

			

**MELANOMA KNOWLEDGE**			

**Which of the following 12 factors increase melanoma risk?^1^**	Correct **(+1)**, don't know **(0)**, incorrect **(0)**	0 to 12	

**How worried would you be if a mole?^2^**	Correct **(+1)**, don't know **(0)**, incorrect **(-1)**	-3 to 6	

**Can melanoma be?^3^**	Correct **(+1)**, don't know **(0)**, incorrect **(-1)**	-4 to 4	

**Commonest site of melanoma in men or women?^4^**	Correct **(+1)**, don't know **(0)**, incorrect **(-1)**	0 to 1	**-7 to 23**

^1 ^having lots of moles, a particular diet, fair complexion, alcohol, sunburn, prolonged sun exposure, smoking, blue eyes, green eyes, red hair, fair hair, dark hair

^2 ^became irregular in shape, became irregular in colour, grew in size

^3 ^completely prevented, heal without treatment, be cured in treated early, lead to death if not treated

^**4 **^Face, back, leg, other (specify), don't know

Please note that questions appearing in the table have been abridged

### Data handling

Paper questionnaires were manually coded prior to entry into a Microsoft Access database. Data were subsequently exported into SPSS version 17.0 for statistical analysis.

### Outcome measures

Outcome measures of the questionnaire are the four summary scores: protective behaviour, personal risk, level of concern and melanoma knowledge.

### Statistical analysis

All statistical analysis was conducted using SPSS 17.0. Demographic variables were summarised using percentage (n) or mean (standard deviation) and compared across nationalities using either the chi square test or the unpaired t-test. In univariate analysis, since three of the summary scores (personal risk, protective behaviour and knowledge) were Normally distributed, they were compared between groups using the unpaired t-test. The other (level of concern) was non-Normally distributed and levels were compared using the Mann-Whitney U test. Multiple linear regression was used to adjust for gender, nationality, age, having a diagnosis of skin cancer, personally knowing someone with skin cancer, and educational status with each of the four summary scores (personal risk, level of concern, behaviour and knowledge scores) as the dependent variables.

## Results

The demographic characteristics of those completing the questionnaire are summarised in table 2. The Scottish respondents were significantly older (p = 0.007) and of lower educational status (p < 0.001) compared with the Australian respondents. Significantly more of the Australian group personally knew someone with skin cancer (p < 0.001).

**Table 2:** Comparison of demographic characteristics between the Scottish and Australian participants

	Scottish	Australian	P-value
**Gender**			

Male	127 (46.5)	115 (43.1)	

Female	146 (53.5)	152 (56.9)	0.420

			

**Age mean (SD)**	56.8 (15.2)	53.2 (15.5)	0.007

			

**Educational status**			

No qualifications	46 (17.7)	2 (0.7)	

Basic school qualifications	53 (20.4)	10 (3.7)	

Higher school qualifications	16 (6.2)	100 (37.5)	

Vocational	37 (14.2)	83 (31.1)	

Degree/professional	108 (41.5)	72 (27.0)	<0.001

			

**Diagnosed with skin cancer**			

Yes	133 (48.7)	83 (31.1)	

No	140 (51.3)	184 (68.9)	<0.001

			

**Personally know someone with skin cancer**			

Yes	120 (44.0)	190 (71.2)	

No	153 (56.0)	77 (28.8)	<0.001

Values are n (%) unless otherwise stated P-values from chi squared test

Table 3 summarises the univariate comparison of the four summary scores by nationality. There was no difference between Australians and Scots in personal risk score. The median level of concern summary score was significantly higher among the Australian participants (p < 0.001), as were the mean summary scores for protective behaviour (p < 0.001) and knowledge (p < 0.001). Tables 4 and 5 summarises data by a personal diagnosis of skin cancer (melanoma and non-melanoma skin cancer), and knowing someone personally with melanoma. Both Australians and Scots previously diagnosed with skin cancer (melanoma and non-melanoma skin cancer) had significantly higher personal risk and level of concern than their non-affected compatriots. In terms of protective behaviour there was no significant difference between affected and non-affected Australians, whereas Scots previously diagnosed with melanoma reported greater protective behaviour than non-affected Scots. Conversely, Australians previously diagnosed with skin cancer (melanoma and non-melanoma skin cancer) had greater knowledge than Australians not affected, but there was no significant difference between Scots irrespective of a prior melanoma diagnosis. Australians and Scots who knew someone else that had been affected by melanoma had significantly higher personal risk scores and knowledge than those who did not know someone affected with melanoma. Knowing someone with melanoma did not appear to significantly increase level of concern or protective behaviour in either Scots or Australians.

**Table 3:** Univariate comparison of the four summary scores by nationality

	Mean (SD) or median (IQR)	P-value
**PERSONAL RISK**		

**Scottish**	11.37 (3.78)	

**Australian**	11.58 (3.66)	0.517

		

**LEVEL OF CONCERN**		

**Scottish**	3.00 (0.00 to 5.00)	

**Australian**	6.00 (3.00 to 8.00)	<0.001

		

**PROTECTIVE BEHAVIOUR**		

**Scottish**	11.91 (3.23)	

**Australian**	13.64 (2.65)	<0.001

		

**KNOWLEDGE**		

**Scottish**	11.90 (4.21)	

**Australian**	13.87 (3.14)	<0.001

P-values from unpaired t-test or Mann-Whitney U test

**Table 4:** Univariate comparison of the four summary scores by personal diagnosis of skin cancer

			Mean (SD) or Median (IQR)	P value
**PERSONAL RISK**	**Australian**	**Previous skin cancer**	12.30 (3.35)	
		
		**No previous skin cancer**	11.25 (3.75)	0.024
		
				
	
	**Scottish**	**Previous skin cancer**	12.59 (3.60)	
		
		**No previous skin cancer**	10.22 (3.60)	<0.001
		
				

**LEVEL OF CONCERN**	**Australian**	**Previous skin cancer**	9.00 (6.50 to 11.00)	
		
		**No previous skin cancer**	5.00 (3.25 to 9.00)	<0.001
		
				
	
	**Scottish**	**Previous skin cancer**	4.00 (3.00 to 6.00)	
		
		**No previous skin cancer**	2.00 (0 to 4.00)	<0.001
		
				

**PROTECTIVE BEHAVIOUR**	**Australian**	**Previous skin cancer**	13.95 (2.69)	
		
		**No previous skin cancer**	13.50 (2.62)	0.202
		
				
	
	**Scottish**	**Previous skin cancer**	12.96 (2.87)	
		
		**No previous skin cancer**	10.93 (3.26)	<0.001
		
				

**KNOWLEDGE**	**Australian**	**Previous skin cancer**	14.47 (2.99)	
		
		**No previous skin cancer**	13.60 (3.18)	0.032
		
				
	
	**Scottish**	**Previous skin cancer**	12.32 (4.20)	
		
		**No previous skin cancer**	11.51 (4.20)	0.115

P-values from unpaired t-test or Mann Whitney U test

**Table 5:** Univariate comparison of summary scores by personal knowledge of someone with skin cancer

			Mean (SD) or Median (IQR)	P value
**PERSONAL RISK**	**Australian**	**Knew someone with melanoma**	11.97 (3.39)	
		
		**Not know someone with melanoma**	10.61 (4.11)	0.007
		
				
	
	**Scottish**	**Knew someone with melanoma**	12.33 (3.22)	
		
		**Not know someone with melanoma**	10.61 (4.02)	<0.001
		
				

**LEVEL OF CONCERN**	**Australian**	**Knew someone with melanoma**	6.00 (3.00 to 8.00)	
		
		**Not know someone with melanoma**	4.00 (3.50 to 7.50)	0.172
		
				
	
	**Scottish**	**Knew someone with melanoma**	4.00 (2.00 to 5.00)	
		
		**Not know someone with melanoma**	3.00 (0 to 5.00)	0.071
		
				

**PROTECTIVE BEHAVIOUR**	**Australian**	**Knew someone with melanoma**	13.66 (2.71)	
		
		**Not know someone with melanoma**	13.58 (2.49)	0.875
		
				
	
	**Scottish**	**Knew someone with melanoma**	11.82 (3.08)	
		
		**Not know someone with melanoma**	11.99 (3.36)	0.662
		
				

**KNOWLEDGE**	**Australian**	**Knew someone with melanoma**	14.07 (3.14)	
		
		**Not know someone with melanoma**	13.38 (3.11)	0.001
		
				
	
	**Scottish**	**Knew someone with melanoma**	12.82 (3.88)	
		
		**Not know someone with melanoma**	11.17 (4.33)	0.001

P-values from unpaired t-test or Mann Whitney U test

Multivariate regression analysis was then conducted to explore the effect of potential modifiers. In each case nationality, age, sex, educational status, a previous diagnosis of skin cancer (melanoma and non-melanoma skin cancer), and knowing someone else with melanoma were entered into the model (Table 6). Total model R^2 ^values were 0.191 for personal risk summary score; 0.303 for level of concern summary score; 0.179 for protective behaviour summary score, and 0.163 for knowledge summary score. Of factors that independently predicted a higher personal risk summary score, younger age, having a diagnosis of skin cancer (melanoma and non-melanoma skin cancer), personally knowing someone with melanoma and a higher level of education were all independent predictors. Neither gender nor nationality significantly predicted personal risk summary score. However, Australian nationality was an independent predictor of a higher level of concern summary score, along with having a previous diagnosis of skin cancer (melanoma and non-melanoma skin cancer), a higher level of education and lower age. Gender and personally knowing someone with melanoma did not independently predict the level of concern summary score. Australian nationality was an independent predictor of a high protective behaviour summary score, alongside older age, having had a diagnosis of skin cancer (melanoma and non-melanoma skin cancer) and a higher level of education. A higher level of education was an independent predictor of a high knowledge summary score, along with Australian nationality, personally knowing someone with skin cancer, having had a diagnosis of skin cancer (melanoma and non-melanoma skin cancer) and female gender. Age was not a significant predictor of knowledge summary score.

**Table 6:** Multiple linear regression of each summary score following adjustment for potential confounders (including age, gender, educational status, personal diagnosis of skin cancer, personally knowing someone with skin cancer)

Score	Unstandardised B coefficient (Standard error)	P-value	Total R^2 ^of model
**PERSONAL RISK**			

**Nationality**	-0.045 (0.312)	0.277	

**Age**	-0.299 (0.011)	<0.001	

**Gender**	0.033 (0.303)	0.410	

**Educational status**	0.102 (0.122)	0.017	

**Diagnosed with skin cancer**	-0.286 (0.291)	<0.001	

**Personally knowing someone with melanoma**	-0.191 (0.321)	<0.001	0.191

			

**LEVEL OF CONCERN**			

**Nationality**	0.388 (0.240)	<0.001	

**Age**	-0.105 (0.008)	0.010	

**Gender**	-0.022 (0.233)	0.551	

**Educational status**	0.103 (0.094)	0.009	

**Diagnosed with skin cancer**	-0.368 (0.222)	<0.001	

**Personally knowing someone with melanoma**	-0.053 (0.247)	0.178	0.303

			

**PROTECTIVE BEHAVIOUR**			

**Nationality**	0.316 (0.255)	<0.001	

**Age**	0.188 (0.009)	<0.001	

**Gender**	0.039 (0.248)	0.332	

**Educational status**	0.107 (0.100)	0.013	

**Diagnosed with skin cancer**	-0.218 (0.236)	<0.001	

**Personally knowing someone with melanoma**	0.060 (0.263)	0.159	0.179

			

**KNOWLEDGE**			

**Nationality**	0.194 (0.320)	<0.001	

**Age**	-0.012 (0.011)	0.788	

**Gender**	0.109 (0.311)	0.008	

**Educational status**	0.224 (0.126)	<0.001	

**Diagnosed with skin cancer**	-0.150 (0.296)	<0.001	

**Personally knowing someone with melanoma**	-0.103 (0.330)	0.016	0.163

## Discussion

### Summary of main findings

Univariate comparison of personal risk of melanoma scores by nationality revealed no difference between those Scots and Australians completing the questionnaire. In contrast, Australians' were more concerned about developing skin cancer, reported higher levels of protective behaviour and knew more about skin cancer than Scots and these differences remained following adjustment for other potential confounders. These results are what we expected to find and, therefore, appear to support the content validity of the questionnaire. Of note, the adjusted analysis suggests that in this sample, even Scots diagnosed and treated for melanoma knew less and reported less protective behaviour than unaffected Australians. Again, given the prevailing cultures in the two countries this is unsurprising, albeit somewhat concerning if should it be reproduced in a population study. The adjusted analysis also reveals that in this convenience sample, as expected, being diagnosed with any type of skin cancer increased concern, protective behaviour and knowledge. Knowing someone else diagnosed with melanoma had a lesser effect leading to increased concern and knowledge, but not self reported protective behaviour. Again, considering the convenience samples used these results are what we should have expected to find.

### Context with other literature

We report a study seeking to confirm the content validity of a questionnaire in a convenience sample of participants and any comparisons with other literature must be correspondingly tempered and cautious. Nevertheless, the findings are consistent with reports that primary prevention efforts have succeeded in Australia, especially amongst younger people where increases in incidence appear to be levelling off[3]. Conversely, the view that primary prevention and education are currently suboptimal in Scotland are supported by the data, and is consistent with the continued rise in incidence here[10]. Two studies, conducted since the introduction of SunSmart in the UK in 2003, which lend further credence to our questionnaire should be mentioned here[6,7]. Both found that young British men of lower socioeconomic and educational status knew least about skin cancer, whereas women, the elderly and those at highest risk were most likely to be concerned about it. They also reported sub-optimal practice of sun protection, although women, older people, those of higher educational status and those at highest risk appeared to be most proactive. Most worryingly, in 2005, 65% of respondents associated a sun tan with good health[6]. Perhaps in relation to this, Diffey et al (2009) reported that British people generally tend to underestimate their personal risk of skin cancer, and despite high levels of concern, as indicated by high levels of self reported self examination, continue to practice high risk activities such as sun-bathing without sunscreen, particularly younger people[7]. Although the data on self examination were encouraging, taken together these findings are all the more concerning since those taking part had a personal interest in skin cancer due to the recruitment method[7]. Both studies correspond closely to the findings presented here, further supporting the content validity of the questionnaire. Neither study explored the effect of personal experience (either having or knowing someone with skin cancer) on knowledge and behaviour. Our findings suggest that these are important and may point the way toward how educational strategies could be designed in future.

### Strengths and limitations

We are quite clear that the purpose of this pragmatic study was to provide evidence of the content validity of a questionnaire to measure personal risk, knowledge and protective behaviour with respect to melanoma. We can make no claims beyond this. Participants were drawn purposively from groups between which we would have expected to have found differences in these domains. In this respect, we believe that the data appear to show the questionnaire can detect these differences. Furthermore, we believe that this has been an important exercise, since the way in which the questionnaire is designed, with multiple response items which vary the number of responses which people can assign, mean that it would not be valid to conduct conventional tests of internal consistency. Further, the questionnaire appears to have performed well amongst both Scottish and Australian people. In both populations it appears to have been understood and easily completed with very little missing data, despite a wide range of demographic characteristics. The questionnaire had been used before but the way we have used and analysed it, in two discrete geographical regions and computing four separate summary scores is novel. We believe that this method of analyzing the questionnaire can provide meaningful information on separate domains of a persons' risk of and attitudes to skin cancer and supports it use in future Scottish studies to determine where melanoma prevention advice should be targeted and subsequently evaluated.

Several limitations must be acknowledged. First and foremost, the four study groups were formed purposively as described. Therefore, although our data makes intuitive sense, we make no claims as to its external validity and generalizability to the general populations of both countries. That was not our purpose in conducting this study.

It would have been logical to have explicitly included a sample of Australian people treated for melanoma. We did not do this due to a concern that Australian people with melanoma are an over-researched group.

Data was not collected from all groups at the same time. Data was collected from Scottish melanoma patients about two years before the other Scottish group, with Australian data collection occurring at approximately the same time. Arguably, therefore this could mean that differences between the Scottish groups could have been attenuated by publicity and prevention campaigns in the interim, although differences were still striking. Also, of the people affected by skin cancer, all of the Scots had had melanoma, whereas the affected Australians comprised those affected by all skin cancers. If anything, this should have biased the study toward the Scots. With the caveat of the previously discussed methodological limitations the differences we demonstrate could, therefore, considerably underestimates the true values within the Scottish population.

### Implications

Our data imply that the questionnaire used in this study has potential as a research tool to guide melanoma prevention efforts, certainly in Scotland. In future, and in carefully designed population studies, it could be administered to large numbers of people. The data thus generated could then be used to target resources for melanoma prevention in the most appropriate way. Firstly, baseline personal risk domain data could identify population groups with differential personal risk thereby allowing efforts to be targeted. In conjunction with baseline population data on level of concern, melanoma knowledge and self reported protective behaviour melanoma prevention efforts could be directly targeted where it is most needed. These data could also permit prevention efforts to be subtly tailored to achieve the maximum impact. Secondly, once prevention had been implemented and after a suitable period of time the questionnaire could be used to assess the impact of efforts, allowing subsequent redirection of effort if needed.

## Conclusions

We have demonstrated that the questionnaire described provides meaningful data with respect to melanoma risk, concern, knowledge and self-reported protective behaviour and could be useful in future to target and assess the impact of melanoma prevention efforts.

## Competing interests

The authors declare that they have no competing interests.

## Authors' contributions

PM designed the study. JE and TW co-ordinated data collection in Western Australia, PM conducted data collection in northeast Scotland. HG conducted the analysis of the data under the direction of AL. HG and PM wrote the paper with comments on drafts from JE, TW and AL. All authors have read and approved the final manuscript.

## Pre-publication history

The pre-publication history for this paper can be accessed here:

http://www.biomedcentral.com/1471-2288/11/123/prepub
